# Hesitancy towards COVID-19 Vaccination among Healthcare Workers: A Multi-Centric Survey in France

**DOI:** 10.3390/vaccines9060547

**Published:** 2021-05-22

**Authors:** Cécile Janssen, Alexis Maillard, Céline Bodelet, Anne-Laure Claudel, Jacques Gaillat, Tristan Delory, on behalf of the ACV Alpin Study Group

**Affiliations:** 1 Centre Hospitalier Annecy Genevois, Infectious Diseases Unit, F-74374 Annecy, France; cjanssen@ch-annecygenevois.fr; 2 Centre Hospitalier Annecy Genevois, Clinical Research Unit, F-74374 Annecy, France; maillard.alexis@laposte.net (A.M.); alclaudel@ch-annecygenevois.fr (A.-L.C.); jgaillat@ch-annecygenevois.fr (J.G.); 3 Laboratoire Inter-universitaire de Psychologie (LIP-PC2S), Université Grenoble, Alpes 1251 Avenue Centrale, 38400 Saint-Martin-d’Hères, France; celine.bodelet@univ-grenoble-alpes.fr

**Keywords:** COVID-19, vaccination, hesitancy, healthcare workers, cross-sectional survey, clustering, unsupervised learning

## Abstract

Vaccination programs against COVID-19 are being scaled up. We aimed to assess the effects of vaccine characteristics on vaccine hesitancy among healthcare workers in a multi-center survey conducted within French healthcare facilities from 1 December 2020 to 26 March 2021. We invited any healthcare workers naïve of COVID-19 vaccination to complete an online self-questionnaire. They reported on their socio-demographic characteristics, as well as their perception and beliefs towards vaccination. We measured their willingness to get vaccinated in eight scenarios for candidates’ vaccines presented sequentially (1 to 4-point scale). Candidates’ vaccines varied for efficacy (25%, 50%, 100%), length of immunization (1 year or lifetime), frequency (<1/100, <1/10,000), and severity (none, moderate, severe) of adverse events. We analyzed 4349 healthcare workers’ responses with interpretable questionnaires. The crude willingness to get vaccinated was 53.2% and increased over time. We clustered the trajectories of responses using an unsupervised classification algorithm (k-means) and identified four groups of healthcare workers: those willing to get vaccinated in any scenario (18%), those not willing to get vaccinated at all (22%), and those hesitating but more likely to accept (32%) or reject (28%) the vaccination depending on the scenario. In these last two subgroups, vaccine acceptance was growing with age, educational background and was higher among men with condition. Compared to an ideal vaccine candidate, a 50% reduced efficacy resulted in an average drop in acceptance by 0.8 (SD ± 0.8, −23.5%), while it was ranging from 1.4 (SD ± 1.0, −38.4%) to 2.1 (SD ± 1.0, −58.4%) in case of severe but rare adverse event. The acceptance of a mandatory immunization program was 29.6% overall and was positively correlated to the willingness to get vaccinated, ranging from 2.4% to 60.0%. Even if healthcare workers represent a heterogeneous population, most (80%) could accept the vaccination against COVID-19. Their willingness to get the vaccine increased over time and as immunization programs became available. Among hesitant professionals, the fear of adverse events was the main concern. Targeted information campaigns reassuring about adverse events may increase vaccine coverage, in a population with a strong opinion about mandatory immunization programs.

## 1. Introduction

The massive scaling-up of immunization programs is essential to tackle the COVID-19 pandemic and progressively reduce the burden of bundles of preventive measures [1].

On the frontline, healthcare workers have a high risk of infection and are a key population for vaccination, as their protection serves the preservation of the healthcare system [2,3,4,5]. A high coverage among these professionals could also increase vaccination acceptance within the general population.

However, during the last 2009 A(H1N1) influenza pandemic, the global vaccine coverage remained below 50% among healthcare workers [6,7]. In Europe, the coverage for seasonal influenza vaccines ranges from 27.5% in Spain to 54.7% in UK-Wales (2018) [8]. It is also known that healthcare workers are a heterogeneous population in their attitudes towards vaccination in general [9]. In France, the 2018–2019 coverage for flu among caregivers working in a hospital was 34.8% and varied by area, occupation, age, and sex [10].

To decipher COVID-19 vaccine hesitancy and highlights its determinants, we conducted a multi-centric survey evaluating vaccine acceptance for different vaccine candidates among healthcare workers of French institutions.

## 2. Materials and Methods

### 2.1. Study Context, Design, and Population

We conducted a multi-centric cross-sectional survey among healthcare workers of French institutions, including public hospitals, private hospitals, and nursing homes. Twenty-one chief executive officers of French healthcare facilities agreed to participate in the study. Any participating institution had to broadcast to their staff members a QR-code and a web-link, by e-mail, notice on the intranet of institutions, hanging of advertising posters within the institution, or adding an advertising notice to the monthly pay slip. These links were redirecting to an online self-questionnaire (supplementary materials). Data were collected from 1 December 2020 to 26 March 2021. On 16 March 2021, we contacted the participating sites to broadcast a reminder to their staff members.

The national immunization campaign for French healthcare workers started on 4 January, with the scaling-up of the BNT162b2 mRNA vaccine (Pfizer-BioNTech). Initially, any healthcare worker aged 50 and older was eligible for vaccination. From 6 February to 15 March 2021, those aged 50 and below were eligible for the AZD1222 adenovirus vaccine (AstraZeneca). From the 15 March to the 19 March, following an European Medicines Agency warning, French health authorities temporarily banned the AZD1222 adenovirus vaccine for safety reasons [11]. It was then re-authorized for staff aged 55 and above only. At the same date, all healthcare workers became eligible for the BNT162b2 mRNA vaccine.

### 2.2. Self-Questionnaire

A unique quick response (QR)-code and web link to reach the self-questionnaire were provided to each institution. Data collected through the online self-questionnaire were anonymous. After flashing the QR-code or clicking the web link, employees had to report their vaccine status towards COVID-19.

### 2.3. Scenario of Candidate Vaccines

Each scenario was characterized by different vaccine efficacy (25%, 50%, 100%), length of immunization (1 year, lifetime), frequency (<1/100, <1/10,000), and severity of the induced adverse event (none, moderate, severe). In any scenario, we hypothesized that COVID-19 epidemic would become annual and seasonal. Scenarios were sequentially ordered from first to last. The first scenario was an ideal vaccine candidate with 100% efficacy, lifetime immunization, and no induced adverse events. Scenarios are detailed in Supplementary Table S1.

### 2.4. Outcomes/Statistical Analysis

We first estimated the crude willingness to get vaccinated against COVID-19 vaccination and over time (in weeks). Then we estimated the evolution of the willingness towards COVID-19 vaccine through scenario analysis, starting from an ideal vaccine candidate (first scenario). We considered the sequential measurement of willingness as a longitudinal array, corresponding for each respondent, to a trajectory of vaccine acceptance. For complete cases, we clustered individual trajectories to identify subgroups of attitudes towards vaccine acceptance using the measurement of temporal Euclidean distance (k-means for longitudinal data) [12]. Based on the Elbow method, we determined that four clusters would explain ~80% of the variance: (1) those willing to be vaccinated at any cost; (2) those hesitating but likely to get vaccinated; (3) those hesitating but not likely to get vaccinated; and (4) those not willing to get vaccinated at all. We used frequencies (percentages), and median (interquartile range) to describe these four clusters. Among hesitant healthcare workers, we estimated the conditions which negatively impacted vaccine acceptance: efficacy (100% to 50%), adverse event severity (none to moderate, to severe), or length of immunization (lifetime to 1 year). We finally compared potential leverages to increase willingness to get vaccinated by comparing healthcare workers’ beliefs about COVID-19 vaccination. We used the chi-squared test and the Fischer exact test to compare frequencies between clusters. We also used the Student t-test and the Mann–Whitney test to compare distributions. We set the level of significance to 5% bilateral (*p*-value < 0.050). All analyses were performed on the R software version 4.0.3 (The R Project for Statistical Computing, Vienna, Austria). We used the ‘kml’ and ‘ggplot2′ packages.

## 3. Results

**Figure 1:**
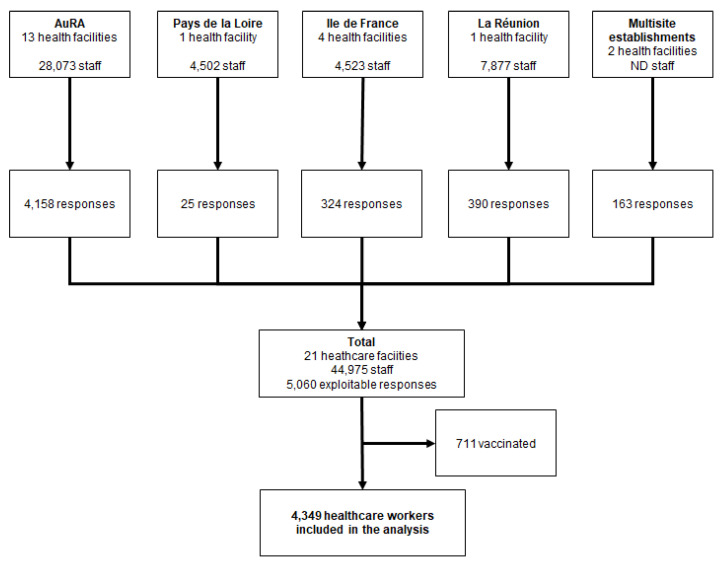
Study flow chart. ND—not-defined.

Over the study period, more than 45,000 workers from 21 healthcare facilities were reached, of whom 8773 opened the online survey: 711 were vaccinated and were therefore excluded from clustering analyses, 4349 had exploitable responses, and were thereby included in the analyses (Figure 1). The sample was mainly composed of women (74.4%), aged 25 to 50 (71.3%), with a broad range of educational levels, and a third having a master’s degree or higher. Half of the participants were acting as frontline caregivers, including 31.8% nurses and nurse assistants and 14.4% physicians. The majority (86.7%) were working in public hospitals, and the remaining in private facilities or nursing homes.

**Figure 2:**
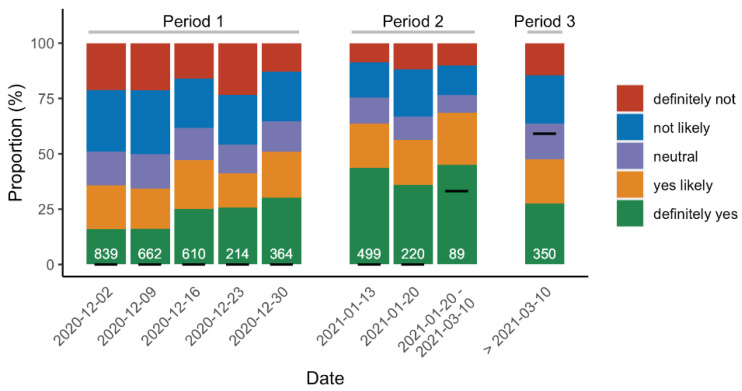
Time course of raw-willingness score to be vaccinated against COVID-19. White numbers represent the number of participants enrolled each week (poll time points). Participants were categorized in 5 groups according to their self-reported crude willingness to be vaccinated against COVID-19 (1 to 7 scale): definitely, yes (7), yes, likely (5–6), neutral (4), not likely (2–3), definitely, not (1). Black bars show the proportion of vaccinated respondents. Period 1: no vaccine available. Period 2: BNT162b2 mRNA vaccine available for healthcare workers >50 years and AZD1222 adenovirus vaccine for those <50 years. Period 3: BNT162b2 mRNA vaccine for any healthcare worker, and AZD1222 adenovirus vaccine for those >55 years only (after a temporary ban for safety concerns).

The crude willingness to get vaccinated against COVID-19 (including already vaccinated respondents) was 53.2% overall (4,558 responses), including 15.6% vaccinated, 21.8% definitely, yes, and 16.8% yes, likely. Others were neutral (11.9%), not likely (19.8%), or not willing to get vaccinated at all (14.1%). After the start of the immunization program, the proportion of respondents of the poll vaccinated against COVID-19 reached 59.1% after 10 March 2021. As illustrated in Figure 2, the willingness increased over the study period (*r*^2^ = 0.010, *p*-value < 0.001).

### 3.1. Scenario and Vaccine Acceptance

**Figure 3:**
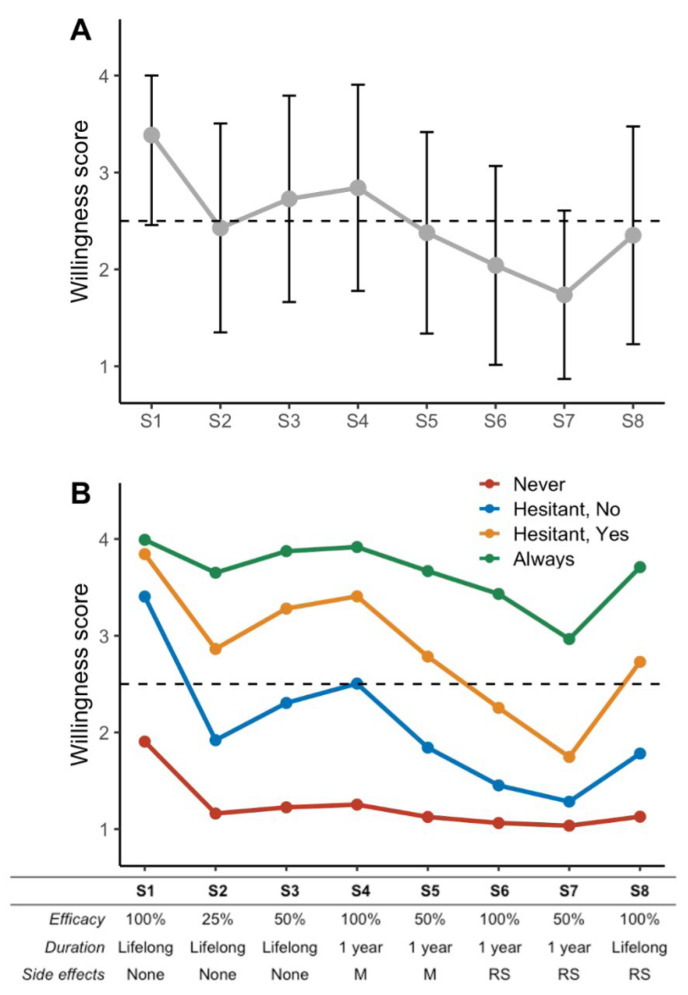
Trajectory of responses to vaccine scenarios and clustering. Eight vaccine candidate scenarios were submitted to participant in the same order from #1 (S1, ideal vaccine candidate conferring a lifelong immunity with 100% efficacy and no adverse events) to #8 (S8). Vaccine efficacy ranges from 25% to 100%. Immunization duration was 1-year to lifelong. Side effects were absent, moderate (M) or rare but severe (RS). Participant scored (1 to 4 scale) each scenario according to their willingness to be vaccinated with the proposed candidate from 1 (“Not at all”) to 4 (“Definitely”). (**A**) Average trajectory of responses across the scenarios; error bars represent standard-deviations, dashed horizontal line the limit between scores in favor (above) or in disfavor (below) of vaccine acceptance. (**B**) Average trajectories of vaccine acceptance for the four clusters obtained using a k-mean algorithm for longitudinal data (see methods): “Never” group in red, “Always” group in green, “Hesitating but willing” group in yellow and “Hesitating and not willing” group in blue.

We presented eight candidate vaccine scenarios to participants, always in the same order, varying for efficacy, duration of immunity, and adverse events. Complete responses for candidate vaccines were available for 3732 (85.8%) of respondents, which were clustered. Figure 3A shows the evolution (trajectories) of vaccine acceptance over the scenario (scale of 1 to 4, from 1, “Definitely, not” to 4, “Definitely, yes”). The average acceptance decreased from a maximum of 3.4 (SD ± 0.9) with an ideal vaccine candidate (scenario 1) to a minimum of 1.7 (SD ± 0.9) in the worst-case scenario (scenario 7), with the lowest efficacy and the worst side effects.

The k-means algorithm clustered participants into four subgroups for vaccine acceptance (Figure 3B): those with almost constant negative answers (group “Never”), those with almost constant positive answers (group “Always”), and the two remaining hesitant groups despite a trend to rather positive (“Hesitant but willing” group) or negative (“Hesitant but not willing” group) willingness.

### 3.2. Cluster Characteristics

The characteristics of each clusters’ staff members are described in Table 1. Workers not likely or not willing at all to get the vaccine were the youngest, more frequently women, less educated, and working in administrative functions with a relatively lower income. They also had a low coverage for mandatory vaccines—hepatitis B, combined (diphtheria, tetanus, poliomyelitis), and whooping cough—and yearly recommended influenza vaccination (17.6% to 42.3% versus 75.3% to 91.1% in others, *p* < 0.001). Exposure to COVID-19 cases and rate of infection was similar across clusters, though the frequency of risk factors for severe COVID-19 infection was higher in those willing to get the vaccine.

As described in Table 2, the fear of adverse events (severity and frequency) was higher in healthcare workers not willing to get vaccinated. Most (51.8% to 58.7%) believed that pharmaceutical companies had a beneficial interest in increasing population vaccination coverage (*p*-value < 0.001). Up to a quarter of them (13.9% to 27.7%) supported that alternative medicine and homeopathy were efficient against COVID-19 (*p*-value < 0.001). Empathy, assessed through five questions, was similar across clusters (Supplementary Figure S1).

### 3.3. Efficacy, Adverse Events and Length of Immunization Variations on Vaccine Acceptance among Hesitant Healthcare Workers

**Figure 4:**
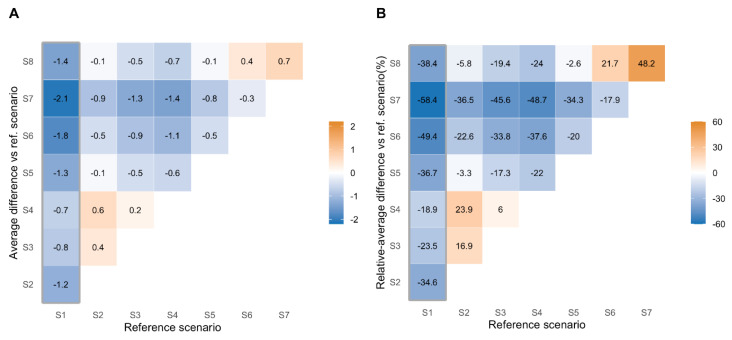
Vaccine willingness across scenarios among hesitating healthcare workers. Absolute (**A**) and relative (**B**) differences of the average score for vaccine-willingness (scale from 1 to 4) between a reference scenario with an ideal vaccine candidate (abscissa) and subsequent scenarios (ordinate) among hesitating 2240 participants (**A**). In (**B**), the relative percentage of variation in mean difference is given.

Figure 4 displays the raw and relative differences in vaccine acceptance by scenario. Among hesitant healthcare workers, the scenario analysis revealed that any decrease in vaccine efficacy or length of immunization resulted in a decrease in vaccine acceptance. However, the strongest and most negative effect was observed for the situation in which adverse events severity was increasing, even if rare (Supplementary Figure S2).

Indeed, the combination of decreased efficacy, one-year immunization, and severe adverse events (scenario 7) lead to a 2.1 drop (SD ± 0.8) in vaccine acceptance from the ideal (−58.4%). Moreover, in such conditions, the decrease in efficacy from 100% to 50% (scenario 6) only resulted in a 0.3 drop (SD ± 0.6, −17.9%) in vaccine acceptance. Finally, when switching from an ideal to a vaccine candidate with severe adverse events (scenario 8) the vaccine acceptance dropped by 1.4 (SD ± 1.0, −38.4%), whereas moderate but frequent adverse events (scenario 4) only lead to a 0.7 drop (SD ± 0.8, −18.9%).

**Figure 5:**
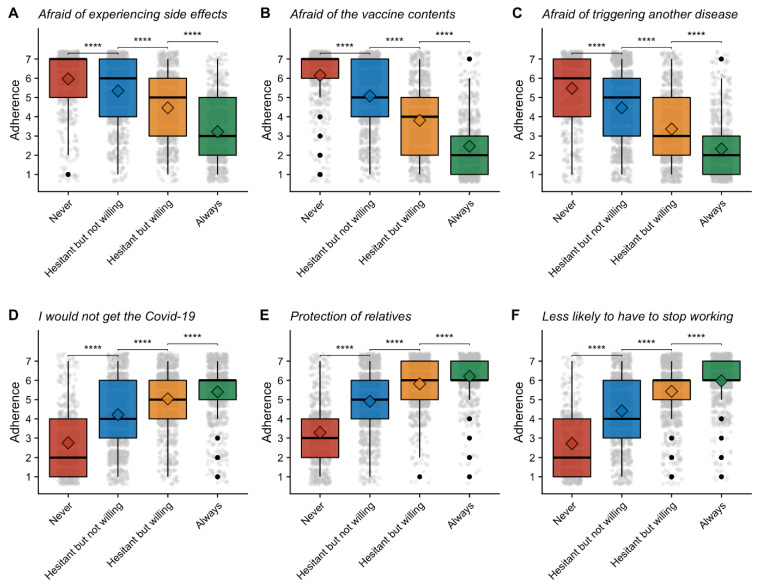
Perception of vaccination against COVID-19 within each cluster. Boxplots of the intensity of perceptions and beliefs about COVID-19, ranging from 1 (“Not at all”) to 7 (“Absolutely”). Black dot are representing outliers and greys dots the distribution of answers. For each panel, participants were asked to answer to the question “If you were to be vaccinated against COVID-19…”: (**A**) “…I would be afraid of experiencing side effects” (**B**) “…would be afraid of the contents of the vaccine” (**C**) “…I would be afraid of triggering another disease” (**D**) “…I would not get the virus”, (**E**) “…I would be protecting patients and/or my family”, (**F**) “…I would be less likely to have to stop working”. **** for *p*-value < 0.001, Wilcoxon-test.

Consistently, willingness to get vaccinated was inversely correlated with the fear of COVID-19 vaccine composition and its potential adverse effects on the body (Figure 5A–C). Those with the least vaccine acceptance also had the lowest confidence in vaccine efficacy (Figure 5D–F).

### 3.4. Mandatory Vaccination

Less than a third of healthcare workers were willing to accept mandatory vaccination programs, ranging from 2.4% among those unwilling to get vaccinated to 60% among those willing.

## 4. Discussion

The willingness to get vaccinated against COVID-19 among healthcare workers increased over time and with the availability of vaccines. After clustering, less than a fifth of health professionals appeared to be firmly reluctant towards vaccination, and most are hesitant. Overall, vaccine willingness grew with age, educational background and was higher among men with underlying conditions. The main determinant of vaccine hesitancy seemed to be the fear of adverse events.

Unsupervised classification methods allowed us to investigate healthcare workers as a heterogeneous population and to identify that a majority were hesitant in their willingness to get vaccinated against COVID-19. Compared to multiple regression models, unsupervised clustering measures average perceptions within subgroups having homogeneous profiles of responses to a set of questions though having different responses to individual questions. Therefore, instead of using a subjective self-reported measurement of vaccine acceptance for identifying hesitant healthcare workers, we are using their global attitude towards vaccination. Such approaches are helpful to use perceptions and attitudes of each cluster towards vaccination in cluster targeted-communication campaigns for increasing vaccine acceptance within hesitant staff members.

As such, safety seems to be a genuine concern for healthcare workers, beyond efficacy and length of immunization. This is highlighted by the recent global alert for the AZD1222 adenovirus vaccine. If a choice were to be given, healthcare workers might favor vaccines with the least side effects, including mRNA vaccines [11,13]. Consistently, a recent cohort study conducted among healthcare workers in England showed that 89% of the personnel had been vaccinated with the BNT162b2 mRNA vaccine two months after initiating the immunization program. In that study, the characteristics of unvaccinated personnel were similar to those describing our clusters with the lowest vaccine acceptance [14]. Within our cohort, workers having the least willingness to get vaccinated were also the ones having the lowest confidence in institutions and pharmaceutical companies and an incorrect perception of the efficacy of alternative medicines against COVID-19.

The availability of vaccines is likely to be a driver of acceptance in the context of the deadly epidemic persisting. Indeed, the proportion of vaccinated respondents sharply increased after the generalized access to vaccination started in late January. After the 10 March 2021, 59% of respondents to the poll were already vaccinated against COVID-19, versus 46% in the national surveillance of French healthcare workers [15]. Since mid-2020, 15 studies investigated the willingness, and 3 reported the scaling-up of COVID-19 vaccination among healthcare professionals [14,16,17,18,19,20,21,22,23,24,25,26,27,28,29,30,31,32,33,34,35,36,37,38]. Taken together, we can estimate the overall willingness to get vaccinated at 65% (95CI 56% to 73%) among healthcare workers, with a prediction interval ranging from 18% to 94%, and varying by region of the globe (Supplementary Figure S3).

Additionally, global acceptance of mandatory immunization programs was low in our study and correlated with higher COVID-19 vaccine acceptance. This suggests that a mandatory scaling-up of vaccination could be counter-productive and may lead to rejection, particularly in populations with little inclination for vaccination. Coverage for hepatitis B was high among professionals not willing to get vaccinated at all, but in France, this vaccine is mandatory for newly hired hospital staff members. On the other hand, these personnel had a 17.6% coverage for flu. However, from a pragmatic point of view, it seems unrealistic to assess the eligibility for work of caregivers based on their immunization. Communication is therefore essential and should be adapted to healthcare workers’ profiles to turn hesitancy into acceptance. As suggested by our study, vaccine acceptance could also increase with its availability and accessibility.

If the number of positive safety reports were to be increased in the coming months, one could expect that anxiety and fear towards adverse event would decrease. Other components such as efficacy, and length of immunization, would become more relevant, especially in light of the emergence of variants of concerns and recombining viruses [39,40].

Most (60%) professionals were hesitant towards vaccination. Despite higher flu vaccine acceptance in the United Kingdom, the recent reporting of 89% coverage for the first dose of the BNT162b2 mRNA vaccine in England brings hope that hesitant staff will get vaccinated at some point [14]. To that end, training peers using motivational interviewing techniques could also be a promising technique [41,42].

There are many limitations to this study. First, the sample is relatively small and sampling bias with strong opinions about the vaccine. Despite reminders, less than 15% of the targeted population responded to the survey, and the observed coverage for vaccination can result from a biased selection towards a population likely to accept the vaccine. Additionally, as the self-questionnaire was online and anonymous, we cannot exclude the possibility of duplicated responses. Females represented the majority of our sample, but administrative data describing the workforce of the participating sites showed a similar sex ratio. Nearly two-third received yearly vaccination against influenza, which is higher than usually reported for healthcare workers in France [10]. Most of the respondents were caregivers from public healthcare institutions, while the French National Institute of Statistics and Economies Studies reports that up to 30% of healthcare workers are administrative personnel, and half of the caregivers are from the private sector. Additionally, three-quarters of the staff members surveyed reported having been vaccinated at the last poll time point. This is higher than the actual national coverage, estimated at 46% overall [13]. Access to the vaccine was limited to health care workers over 50 years of age during most of the survey period. Therefore, their willingness to be vaccinated may be poorly estimated. Among unvaccinated personnel, we observed a relative decrease in willingness to get vaccinated after the temporary ban of the AZD1222 adenovirus vaccine (10 March 2021) by the European Medical Agency. Interestingly, the features of case scenario #6 were somehow close to the AZD1222 adenovirus vaccine and were associated in responses to a sharp decrease in vaccine acceptance. However, at the same time, vaccine availability increased, and more than half of the respondents had been vaccinated, inducing a biased measurement for vaccine hesitancy. Second, we did not randomize the scenario assignment, there was no individual follow-up over time, and it was thereby not possible to draw a causal inference. Nevertheless, the sequential ordering of the scenario allowed us to use longitudinal analysis and to identify clusters of respondents based on their trajectories of responses. Finally, vaccine availability will increase over time and possibly lead to an increase in vaccine acceptance. In France, the current trend for the scaling of vaccination is a 0.5% daily linear increase. However, an asymptote could be reached in the near future. In this perspective, continued monitoring of vaccine coverage and acceptance is crucial.

## 5. Conclusions

Healthcare workers are a heterogeneous population, but most (80%) could accept the vaccination against COVID-19. Their willingness to get the vaccine increased over time and with access to immunization programs. Among hesitant professionals, the fear of adverse events is the main concern. Targeted information campaigns reassuring about adverse events may increase vaccine coverage in a population with a strong opinion about mandatory immunization programs.

## Figures and Tables

**Table 1:** Characteristics of respondents by cluster.

Characteristics	Missing	Never	Hesitating But Not Willing	Hesitating But Willing	Always	Overall	*p*-Value
	%	N = 675	N = 1190	N = 1050	N = 817	N = 4349	
**Age group**	16.7						<0.001
<25 years		52 (8.0%)	69 (6.0%)	49 (4.8%)	32 (4.0%)	202 (5.6%)	
25–40 years		338 (52.3%)	522 (45.4%)	473 (46.1%)	342 (42.6%)	1675 (46.2%)	
41–50 years		131 (20.3%)	317 (27.6%)	250 (24.4%)	210 (26.2%)	908 (25.1%)	
>50 years		125 (19.3%)	241 (21.0%)	254 (24.8%)	218 (27.2%)	838 (23.1%)	
**Sex, female**	16.7	531 (82.2%)	951 (82.8%)	800 (78.0%)	524 (65.3%)	2806 (77.4%)	<0.001
**Educational level**	27.4						<0.001
High school diploma or less		67 (13.6%)	89 (9.2%)	37 (4.0%)	16 (2.1%)	209 (6.6%)	
Bachelor’s degree		284 (57.6%)	544 (56.3%)	407 (43.6%)	237 (31.0%)	1472 (46.6%)	
Master or higher		71 (14.4%)	216 (22.3%)	391 (41.9%)	431 (56.3%)	1109 (35.1%)	
Other		71 (14.4%)	118 (12.2%)	99 (10.6%)	81 (10.6%)	369 (11.7%)	
**Professional category †**	16.8						<0.001
Frontline caregiver		289 (44.9%)	551 (48.0%)	573 (55.8%)	527 (65.8%)	1940 (53.6%)	
Other caregiver		180 (28.0%)	357 (31.1%)	301 (29.3%)	180 (22.5%)	1018 (28.1%)	
Administrative and non-caregiver staff		165 (25.7%)	228 (19.9%)	145 (14.1%)	86 (10.7%)	624 (17.3%)	
Unclassified		9 (1.4%)	11 (1.0%)	7 (0.7%)	8 (1.0%)	35 (1.0%)	
**Frontline caregivers**							
Doctors	16.8	9 (1.4%)	63 (5.5%)	177 (17.3%)	273 (34.1%)	522 (14.4%)	<0.001
Nurses	16.8	127 (19.8%)	289 (25.2%)	250 (24.4%)	155 (19.4%)	821 (22.7%)	0.003
Assistant nurse	16.8	130 (20.2%)	125 (10.9%)	56 (5.5%)	18 (2.2%)	329 (9.1%)	<0.001
Other †		23 (3.6%)	74 (6.5%)	90 (8.8%)	81 (10.1%)	268 (7.4%)	<0.001
**Relative income ‡**	51.1	5.9 (SD ± 1.9)	6.4 (SD ± 1.8)	6.6 (SD ± 1.8)	6.8 (SD ± 1.8)	6.4 (SD ± 1.8)	<0.001
**Vaccinated against**							
Flu	16.0	115 (17.6%)	491 (42.3%)	778 (75.3%)	733 (91.1%)	2117 (58.0%)	<0.001
Hepatitis B	16.1	531 (81.2%)	1020 (88.1%)	955 (92.4%)	765 (95.1%)	3271 (89.6%)	<0.001
combined (diphteria/tetanos/polio)	16.2	592 (90.7%)	1110 (96.1%)	987 (95.7%)	758 (94.3%)	3447 (94.6%)	<0.001
whooping cough	16.3	450 (68.9%)	881 (76.3%)	833 (80.9%)	666 (82.8%)	2830 (77.7%)	<0.001
**COVID-19 exposure**							
Contact with COVID-19 patients	6.3	531 (78.7%)	931 (78.2%)	845 (80.5%)	675 (82.6%)	3220 (79.0%)	0.083
Household member with COVID-19	7.2	296 (43.9%)	556 (46.7%)	479 (45.6%)	381 (46.6%)	1849 (45.8%)	0.643
SARS-CoV-2 infection	6.7	124 (18.4%)	211 (17.7%)	198 (18.9%)	142 (17.4%)	738 (18.2%)	0.841
**Predisposing conditions** **to severe COVID-19 ^Ϫ^**	7.3	41 (6.1%)	98 (8.2%)	107 (10.2%)	103 (12.6%)	406 (10.1%)	<0.001
**Household member** **with condition for severe COVID-19**	8.2	145 (21.5%)	283 (23.8%)	226 (21.5%)	167 (20.4%)	872 (21.8%)	0.308

† Other: this category includes residents, midwives, physiotherapists, students; ‡ Relative income was assessed using a semi-quantitative Likert scale ranging from 1 (“I am strongly disadvantaged compared to other staff members”) to 10 (“I am strongly advantaged compared to other staff members”; ^Ϫ^ Any condition among the following: age ≥65 years, cardiovascular disease, complicated diabetes, chronic respiratory disease, obesity, late-stage cirrhosis or active cancer, immunodepression, chronic renal failure <30 mL/min/1.73 m^2^, sickle cell disease, pregnancy at 3rd trimester.

**Table 2:** Perceptions and beliefs towards vaccination, including COVID-19.

Characteristics	Missing	Never	Hesitating But Not Willing	Hesitating But Willing	Always	Overall	*p*-Value
	%	N = 675	N = 1190	N = 1050	N = 817	N = 4349	
**Perceptions and attitude towards vaccination and COVID-19**							
Vaccination can induce severe adverse events	27.3	281 (66.1%)	579 (57.0%)	363 (37.1%)	225 (30.3%)	1448 (45.8%)	<0.001
Vaccine-related adverse events are rare	31.8	135 (20.8%)	514 (47.2%)	575 (70.2%)	327 (80.1%)	1551 (52.3%)	<0.001
It is not necessary to get vaccinated, as others are vaccinated	16.2	52 (8.1%)	61 (5.2%)	17 (1.6%)	7 (0.9%)	137 (3.8%)	<0.001
Non-mandatory vaccines are not important	16.5	82 (12.9%)	122 (10.6%)	57 (5.5%)	19 (2.3%)	280 (7.7%)	<0.001
Pharmaceuticals companies are pushing vaccination in their beneficial interest	33.2	178 (58.7%)	473 (51.8%)	360 (39.3%)	174 (22.5%)	1185 (40.8%)	<0.001
Pharmaceuticals companies are important in the public health perspective	40.4	220 (48.7%)	628 (73.5%)	617 (79.7%)	417 (81.6%)	1882 (72.6%)	<0.001
Vaccine are reducing the natural immunity	17.8	174 (30.4%)	191 (16.6%)	71 (6.8%)	13 (1.6%)	449 (12.6%)	<0.001
Self-vaccination can protect others	61.0	247 (43.6%)	493 (71.3%)	273 (79.4%)	75 (81.5%)	1088 (64.2%)	<0.001
I am afraid of COVID-19	8.3	158 (23.4%)	409 (34.4%)	383 (36.5%)	310 (37.9%)	1325 (33.2%)	<0.001
I am confident in the discovery of effective therapies for COVID-19	8.6	145 (21.5%)	479 (40.3%)	521 (49.6%)	416 (50.9%)	1653 (41.6%)	<0.001
Alernative medicine or homeopathy are efficient against COVID-19	8.8	187 (27.7%)	165 (13.9%)	56 (5.3%)	29 (3.5%)	474 (11.9%)	<0.001
**Mandatory COVID-19 vaccination**							
COVID-19 vaccination should be mandatory for healthcare workers	11.5	16 (2.4%)	185 (15.5%)	416 (39.6%)	490 (60.0%)	1138 (29.6%)	<0.001
COVID-19 vaccination should be mandatory for general population	11.5	10 (1.5%)	138 (11.6%)	275 (26.2%)	333 (40.8%)	787 (20.4%)	<0.001

## Data Availability

Anonymized individual-level data, can be shared with academic researchers for 12 months after publication. A codebook corresponding to data reported in the case report form (supplementary materials) is available. Data requests have to be sent at drci.promotion@ch-annecygenevois.fr. The Study Group will review any request to ensure they are fulfilling with the European General Data Protection Regulation.

## Supplementary Materials

The following are available online at https://www.mdpi.com/article/10.3390/vaccines9060547/s1, Figure S1: Self-perception of own empathy within each cluster, Figure S2: Effect of efficacy, adverse events, and length of immunization on vaccine acceptance among hesitating healthcare workers, Figure S3: Global willingness to get vaccinated towards COVID-19 among healthcare workers. The study questionnaire.
