# Supplementary material for: Hesitancy towards COVID-19 Vaccination among Healthcare Workers: A Multi-Centric Survey in France
Source: Vaccines (Basel). 2021 May 22;9(6):547. doi: 10.3390/vaccines9060547 (PMC8224571; doi:10.3390/vaccines9060547)
Supplement: Supplementary file 1 [file vaccines-09-00547-s001.zip › vaccines-1215377-supplementary.pdf]

SUPPLEMENTARY MATERIALS

Table S1 – Scenarios for vaccine candidates.

**Vaccine candidate**	**1 (ideal)**	**2**	**3**	**4**	**5**	**6**	**7 (worst)**	**8**
Efficacy	100%	25%	50%	100%	50%	100%	50%	100%
Immunization	Lifetime	Lifetime	Lifetime	1-year	1-year	1-year	1-year	Lifetime
Adverse events								
Severity	None	None	None	Moderate †	Moderate †	Severe ‡	Severe ‡	Severe ‡
Frequency	None	None	None	Frequent *(1/100)*	Frequent *(1/100)*	Very rare *(<1/10000)*	Very rare *(<1/10000)*	Very rare *(<1/10000)*

The case-vignette scenarios were answered in a sequential order, starting from one (ideal) to eight; † Moderate adverse events were defined as pain at injecting site, followed by few days of influenza-like illness symptoms, ‡ Severe adverse events were defined as neurological disorders, and/or severe allergic reaction, including Quincke oedema.

Figure S1 – Self-perception of own empathy within each cluster

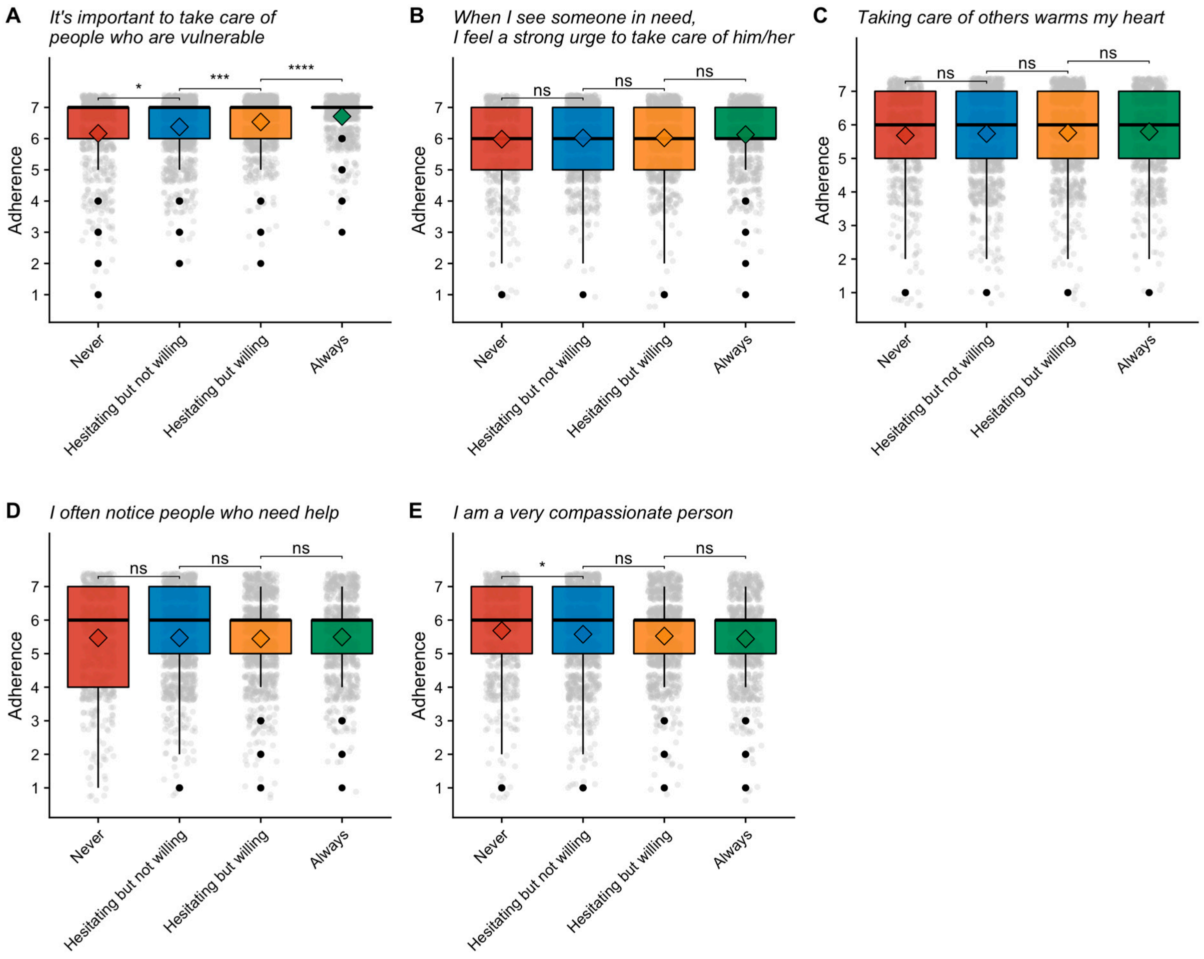

*Boxplots of the intensity of self-perceived own empathy, ranging from 1 ["Not at all"] to 7 ["Absolutely"]. Black dot are representing outliers and greys dots the distribution of answers. For each panel, participants were asked to rate their agreement to following statements: (A) "It's important to take care of people who are vulnerable" (B) "When I see someone injured or in need, I feel a strong urge to take care of them" (C) "Taking care of others warms my heart" (D) "I often notice people who need help" (E) "I am a very compassionate person". ns for non-significant, * for p < 0.05, ** for p < 0.01, *** for p < 0.005, **** for p < 0.001, Wilcoxon-test.*

Figure S2 - Effect of efficacy, adverse events, and length of immunization on vaccine acceptance among hesitating healthcare workers

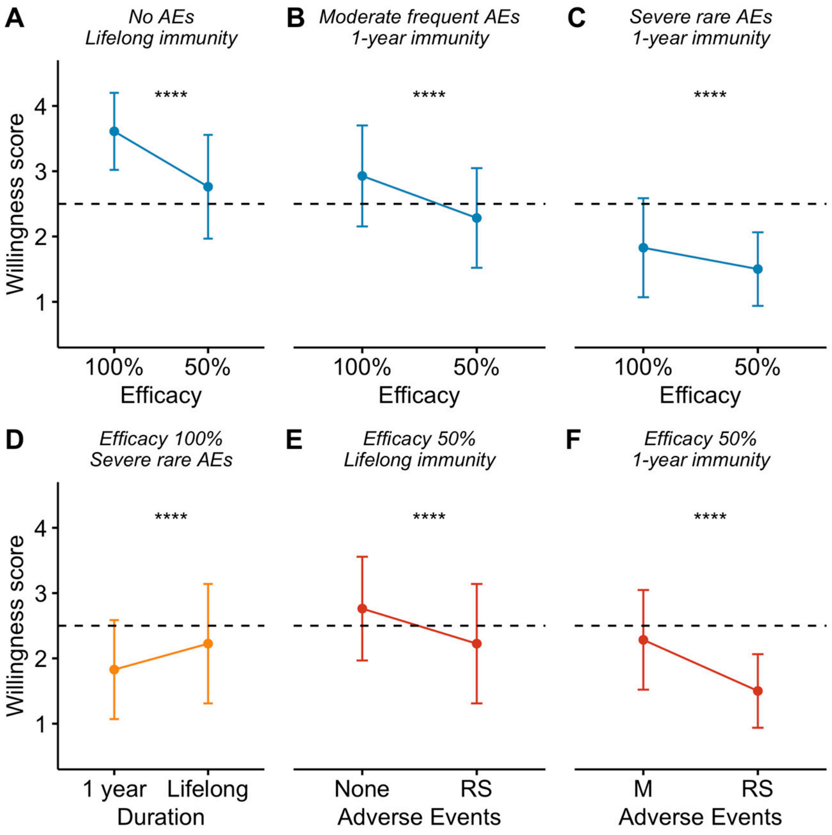

*Among 2,240 hesitating participants: variation of the willingness to get vaccinated according to changes in efficacy (A, B, C), duration of immunity (D), and type of adverse events (E, F). **** for p < 0.001, paired Wilcoxon-test.*

Figure S3 – Global willingness to get vaccinated towards COVID-19 among healthcare workers.

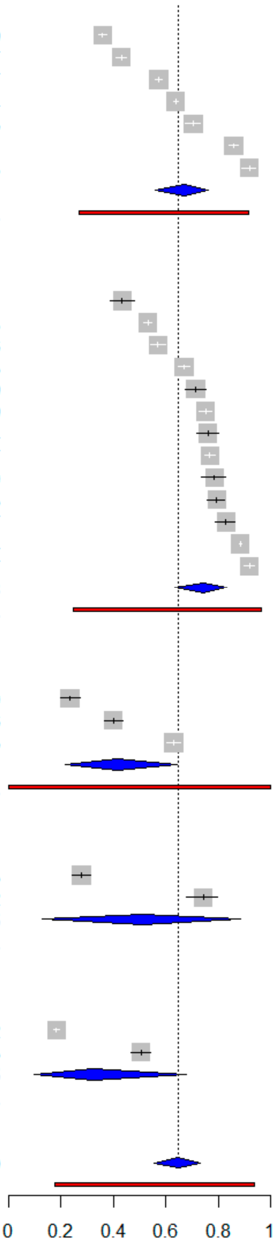

Study	Willing	Surveyed	Willingness	95% CI	Weight
**America**					
Shekhar R et al.	1247	3479	0.36	[0.34; 0.37]	3.7%
Goldman Ran D. et al.	1101	2557	0.43	[0.41; 0.45]	3.7%
Shaw J et al.	3032	5287	0.57	[0.56; 0.59]	3.7%
KuterB.J. et al.	7666	12034	0.64	[0.63; 0.65]	3.7%
Verger P et al.	743	1055	0.70	[0.68; 0.73]	3.7%
Schradling W.A. et al.	1136	1321	0.86	[0.84; 0.88]	3.7%
Amin D.P. et al.	366	398	0.92	[0.89; 0.94]	3.6%
Overall effect	15291	26131	0.67	[0.56; 0.76]	26.0%
Prediction interval	.	.		[0.27; 0.92]	--
Heterogeneity: I^2 = 100%, p = 0					
**Europe**					
Pagagiannis D et al.	200	461	0.43	[0.39; 0.48]	3.7%
Janssen C et al.	2424	4558	0.53	[0.52; 0.55]	3.7%
Nohl A et al.	739	1296	0.57	[0.54; 0.60]	3.7%
Di Gennaro F et al.	1155	1723	0.67	[0.65; 0.69]	3.7%
Dror A.A. et al.	392	549	0.71	[0.67; 0.75]	3.7%
Verger P et al.	910	1209	0.75	[0.73; 0.78]	3.7%
Verger P et al.	315	414	0.76	[0.72; 0.80]	3.7%
Gagneux-Brunon A et al.	1574	2047	0.77	[0.75; 0.79]	3.7%
Pagagiannis D et al.	267	340	0.79	[0.74; 0.83]	3.7%
Robbins T et al.	471	593	0.79	[0.76; 0.83]	3.7%
Szmyd B et al.	321	387	0.83	[0.79; 0.87]	3.7%
Hall VJ et al.	20641	23324	0.88	[0.88; 0.89]	3.7%
Szmyd B et al.	632	687	0.92	[0.90; 0.94]	3.7%
Overall effect	30041	37588	0.74	[0.63; 0.83]	48.1%
Prediction interval	.	.		[0.25; 0.96]	--
Heterogeneity: I^2 = 100%, p = 0					
**Asia**					
Kukreti S et al.	117	500	0.23	[0.20; 0.27]	3.7%
Wang K et al.	322	806	0.40	[0.37; 0.43]	3.7%
Kwok K.O. et al.	759	1205	0.63	[0.60; 0.66]	3.7%
Overall effect	1198	2511	0.41	[0.22; 0.64]	11.1%
Prediction interval	.	.		[0.00; 1.00]	--
Heterogeneity: I^2 = 99%, p < 0.01					
**Africa**					
Kambamba Nzaji M et al.	170	613	0.28	[0.24; 0.31]	3.7%
Kasozi KI et al.	165	222	0.74	[0.68; 0.80]	3.7%
Overall effect	335	835	0.51	[0.13; 0.88]	7.4%
Prediction interval	.	.			--
Heterogeneity: I^2 = 99%, p < 0.01					
**Middle-East**					
Qunaibi E et al.	950	5222	0.18	[0.17; 0.19]	3.7%
Qattan A.M.N. et al.	340	673	0.51	[0.47; 0.54]	3.7%
Overall effect	1290	5895	0.32	[0.10; 0.68]	7.4%
Prediction interval	.	.			--
Heterogeneity: I^2 = 100%, p < 0.01					
**Overall effect**	**48155**	**72960**	**0.65**	**[0.56; 0.73]**	**100.0%**
**Prediction interval**				**[0.18; 0.94]**	
Heterogeneity: I^2 = 100%, p = 0					

We estimated the pooled willingness to get vaccinated towards COVID-19 using the inverse variance method with random effect ('meta' package). Studies reporting vaccine acceptance before the start of immunization programs as well as reporting the scaling of vaccination among healthcare workers were used.

Self-Questionnaire

Healthcare worker vaccinated were excluded from the survey.

Others had to report 1) their demographics; 2) their relative income compared to other staff members; 3) their vaccination history; 4) their perception of COVID-19 disease; 6) their perceptions and attitude towards COVID-19 vaccination; 7) their global empathy; 8) their crude willingness to get vaccinated against COVID-19. Thereafter, they reported their willingness to get vaccinated against COVID-19 for eight virtual vaccine candidates (scenario), using a 4-point Likert scale, ranging from 1 (certainly not) to 4 (certainly yes).

A multidisciplinary committee including infectious disease physicians, epidemiologists, social science specialists, specialists of work-related psychological disorders devised the survey.

It was pilot-tested for clarity, length and validity with 20 healthcare workers.

Groupement Hospitalier de Territoire Alpes Dauphiné · GHT Haute-Savoie Pays de Gex · GHT Savoie-Belley · GHT LEMAN MONT-BLANC GROUPE HOSPITALIER DE TERRITOIRE

ACV ALPIN survey

The ACV ALPIN survey goes national!

At the end of the first wave of the SARS-CoV-2 pandemic, despite a large number of clinical studies, no curative treatment has been discovered, and a preventive vaccine approach seems to be the only solution. Many vaccines are currently under development, and COVID-19 vaccination started in France in January 2021. Healthcare workers, including caregivers, are at high risk of infection because they are in direct or indirect contact with infected persons.

The Centre Hospitalier Annecy Genevois decided to conduct a survey on the COVID-19 vaccination among healthcare workers, initially in the French Alps area, and then throughout the country.

If you work in a health care facility, you are concerned by this survey, regardless of your job position.

This online survey may take up to 15 minutes to complete. We ask you to complete it in one sitting. If you close the survey page, you will not be able to retrieve your previously saved answers. We thank you for participating in this survey only once.

A statistical processing of your answers to the questionnaire will be implemented. These answers will can't allow to identifying you, and are therefore anonymous.

As the questionnaire is completely anonymous, no request for access to your data, rectification or deletion can be taken into account after the questionnaire has been completed, as it will be impossible for us to identify you or link you to your questionnaire.

Your participation is important!

Do not hesitate to tell your colleagues about the survey.

We thank you for your collaboration.

(If you want to participate in the survey, please click on "next")

0. Are you vaccinated against COVID-19?

- Yes
- No, but I am waiting to be vaccinated
- No

If "yes" to question 0 ➔ end of questionnaire, return to page: This questionnaire is now closed. Thank you for your participation.

1. Covid-19 can have serious consequences for my health:

Strongly disagree 1 2 3 4 5 6 7 Strongly agree

2. Have you been in contact with COVID-19 infected patients/persons in the last 6 months?

- Yes
- No
- I don't know

3. Have you had a confirmed Covid-19 infection (positive PCR or positive serology) in the last 6 months?

- Yes
- No
- I don't know

➢ *If « no » to question n°3 :*

4. If you have not had a confirmed Covid-19 infection, how do you estimate the likelihood that you will ever be infected with Covid-19?

- Very likely
- Likely
- Unlikely
- Very unlikely

5. Has anyone close to you had a Covid-19 infection?

- Yes
- No
- I don't know

6. Are you at risk for a severe form of Covid-19?

- Yes
- No
- I don't know

7. If yes, please indicate below which group(s) *you belong* to :

- ≥ 65 years old
- Cardiovascular disease : high blood pressure, severe heart failure, history of stroke, heart attack or heart surgery
- Uncontrolled diabetes or diabetes with complications
- Chronic respiratory disease: chronic obstructive bronchitis, severe asthma, pulmonary fibrosis, sleep apnea, cystic fibrosis, chronic respiratory failure
- Obesity: body mass index > 30 kg/m².
- Advanced cirrhosis
- Progressive cancer under treatment
- Congenital or acquired immunodepression: drug-induced (chemotherapy, immunosuppressive treatment, biotherapy and/or corticosteroid therapy at immunosuppressive dose), uncontrolled HIV infection or with CD4 less than 200/mm3, solid organ or hematopoietic stem cell transplantation, hematologic malignancy under treatment
- Severe chronic renal failure and dialysis
- Syndrome drépanocytaire majeur ou ayant été splénectomisé
- Major sickle cell disease or having been splenectomized
- Third trimester of pregnancy

8. Except yourself, is there any other of your household members at risk for severe Covid-19?

- Yes
- No
- I don't know

9. Do you have a feeling of fear towards Covid-19?

- Yes
- No
- I don't know

10. Are you confident that a cure for Covid-19 will be identified?

- Yes
- No
- I don't know

11. Do you think that alternative medicine (such as vitamins, essential oils, trace elements...) or homeopathy are effective against Covid-19?

- Yes
- No
- I don't know

Please answer the following questions assuming that a vaccine against Covid-19 is available or will be available soon

12. What is your level of interest in the Covid-19 vaccine?

- - I am very interested
- - I am somewhat interested
- - I am not very interested
- - I am not interested at all

13. Who do you think should be recommended the Covid-19 vaccine? (Multiple answers possible)

- To all
- People over 65 years old
- People over 80 years old
- People who are obese
- Infants and children
- People with chronic respiratory disease
- People with chronic heart disease
- People with any chronic disease
- Immunocompromised persons
- People with diabetes
- Health professionnals
- Relatives of health professionals
- Education staff
- Other: ....

14. Getting vaccinated against Covid-19 is for me

Unnecessary	1	2	3	4	5	6	7	Useful
Risky	1	2	3	4	5	6	7	Beneficial
Not recommended	1	2	3	4	5	6	7	Recommended
A weakening	1	2	3	4	5	6	7	A protection

15. Would you be willing to get vaccinated against Covid-19 in a clinical trial?

- Yes
- No
- I don't know

16. Would you be willing to get vaccinated against Covid-19 once clinical trials completed and results available?

- Yes
- No

- I don’t know

17. Do you think that mandatory Covid-19 vaccination is needed for health professionals?
- Yes
- No
- I don’t know

18. Do you think that mandatory Covid-19 vaccination is needed for the general population?
- Yes
- No
- I don’t know

19. Do you intend to get vaccinated against Covid-19?

Not at all 1 2 3 4 5 6 7 Definitely

➢ *<u>If answer to question n°19 > or = 4 (rather AGREE) :</u>*

20. I intend to get the Covid-19 vaccine because (multiple responses possible):
- I am convinced of the importance of vaccination.
- I am one of the people for whom Covid-19 vaccination might be recommended / I am at risk for a severe form
- I am afraid of the possible complications of Covid-19
- I want to avoid transmitting the virus to my relatives (parents, children...).
- I follow the advice of my co-workers.
- I follow the advice of my primary care physician
- I do not want to transmit the virus to the patients/residents I care for in my care unit or nursing home
- Other reason : ……………………………………

➢ *<u>If answer to question n°19 < 4 (rather NOT AGREE) :</u>*

21. I do not intend to get the Covid-19 vaccine because (multiple responses possible):
- I do not feel concerned by this vaccination, even if I am a carrier of the virus.
- I don't trust the vaccine
- I think that vaccination will not be effective
- I fear the side effects of the vaccination.
- I follow the advice of my primary care physician who is against vaccination
- I follow the advice of my co-workers who are against vaccination
- I follow the opinion of social networks that are against vaccination
- I'm afraid the vaccine will give me the disease it's supposed to prevent
- I am afraid of injections
- I have good health in general or I am never sick
- I have a doubt about the content of the vaccine
- Other reason : ……………………………………

22. Eight Covid-19 vaccine candidate scenario will be presented to you. Please answer the question for each vaccine candidate.

<u>Definitions</u>
Percentage of efficacy :
100% = 100% of those vaccinated are protected
50% = 50% of those vaccinated are protected
10% = 10% of those vaccinated are protected

Duration of immunity: duration of protection conferred by the vaccine

In all scenarios, all adverse events are reversible, i.e. they will disappear within a more or less long time after the injection of the vaccine.

Scenario 1
Vaccine efficacy: 100 %
Duration of immunity: for life
Adverse events : None
Evolution of the infection: an epidemic of COVID-19 each year

Do you intend to get vaccinated ?

- Yes, definitely
- Yes, probably
- No, probably not
- No, definitely not

Scenario 2
Vaccine efficacy: 25 %
Duration of immunity: for life
Adverse events : None
Evolution of the infection: an epidemic of COVID-19 each year

Do you intend to get vaccinated ?

- Yes, definitely
- Yes, probably
- No, probably not
- No, definitely not

Scenario 3
Vaccine efficacy: 50 %
Duration of immunity: for life
Adverse events : None
Evolution of the infection: an epidemic of COVID-19 each year

Do you intend to get vaccinated ?

- Yes, definitely
- Yes, probably
- No, probably not
- No, definitely not

Scenario 4
Vaccine efficacy: 100 %
Duration of immunity: 12 months - vaccination must be renewed every year
Adverse events : pain at injection site and flu syndrome for a few days
Evolution of the infection: an epidemic of COVID-19 each year

Do you intend to get vaccinated ?

- Yes, definitely
- Yes, probably
- No, probably not
- No, definitely not

Scenario 5
Vaccine efficacy: 50 %
Duration of immunity: 12 months - vaccination must be renewed every year
Adverse events : pain at injection site and flu syndrome for a few days
Evolution of the infection: an epidemic of COVID-19 each year

Do you intend to get vaccinated ?

- Yes, definitely
- Yes, probably
- No, probably not
- No, definitely not

Scenario 6
Vaccine efficacy: 100 %
Duration of immunity: 12 months - vaccination must be renewed every year
Adverse events : serious adverse reactions (e.g. severe allergic reaction, angioedema, nervous system damage) but very rare (less than 1/10,000)
Evolution of the infection: an epidemic of COVID-19 each year

Do you intend to get vaccinated ?

- Yes, definitely
- Yes, probably
- No, probably not
- No, definitely not

Scenario 7
Vaccine efficacy: 50 %
Duration of immunity: 12 months - vaccination must be renewed every year
Adverse events : serious adverse reactions (e.g. severe allergic reaction, angioedema, nervous system damage) but very rare (less than 1/10,000)
Evolution of the infection: an epidemic of COVID-19 each year

Do you intend to get vaccinated ?

- Yes, definitely
- Yes, probably
- No, probably not
- No, definitely not

Scenario 8
Vaccine efficacy: 100 %
Duration of immunity: for life
Adverse events : serious adverse reactions (e.g. severe allergic reaction, angioedema, nervous system damage) but very rare (less than 1/10,000)
Evolution of the infection: an epidemic of COVID-19 each year

Do you intend to get vaccinated ?

- Yes, definitely
- Yes, probably
- No, probably not
- No, definitely not

23. **Regarding vaccination in general, please indicate how much you agree with the following statements.**

	Strongly agree	Somewhat agree	Somewhat disagree	Disagree	No opinion
Vaccination can cause serious side effects	❑	❑	❑	❑	❑
Adverse events are rare with vaccines	❑	❑	❑	❑	❑
It is not necessary to be vaccinated because many people around us are vaccinated	❑	❑	❑	❑	❑
If a vaccine is not mandatory, it is not that important	❑	❑	❑	❑	❑
Pharmaceutical industries encourage vaccinations to increase profits	❑	❑	❑	❑	❑
Pharmaceutical industries play a role in public health	❑	❑	❑	❑	❑
Vaccines "deplete" the immune system	❑	❑	❑	❑	❑
Vaccinating yourself has an impact on the health of others	❑	❑	❑	❑	❑

24. **How confident are you that it will be easy to get vaccinated against Covid-19?**
Not very confident 1 2 3 4 5 6 7 Very confident

25. **If were to be vaccinated against Covid-19, I would be afraid of having side effects :**
Strongly disagree 1 2 3 4 5 6 7 Strongly agree

26. **If I were to be vaccinated against Covid-19, I would be afraid of the contents of the vaccine**
Strongly disagree 1 2 3 4 5 6 7 Strongly agree

27. **If I were to be vaccinated against Covid-19, I would be afraid of triggering another disease**
Strongly disagree 1 2 3 4 5 6 7 Strongly agree

28. **If I were to be vaccinated against Covid-19, I would not get the virus**
Strongly disagree 1 2 3 4 5 6 7 Strongly agree

29. **If I were to be vaccinated against Covid-19, I would be protecting patients and/or my family.**
Strongly disagree 1 2 3 4 5 6 7 Strongly agree

30. **If I were to be vaccinated against Covid-19, I would be less likely to have to stop working**
Strongly disagree 1 2 3 4 5 6 7 Strongly agree

31. **Are you regularly vaccinated against the flu?**
- - Yes, every year
- - Yes, not every year
- - No
- - I don't know

32. **Have you been vaccinated against hepatitis B?**
- Yes
- No
- I don’t know

33. **Are you up to date with your DTP (diphtheria, tetanus, polio) vaccination?**
- Yes

- No
- I don’t know

34. Are you vaccinated against whooping cough?

- Yes
- No
- I don’t know

35. Do you have confidence in the health authorities regarding vaccine recommendations?

- Yes, a lot
- Yes, a little
- No
- I do not know

36. It's important to take care of people who are vulnerable:

Strongly disagree 1 2 3 4 5 6 7 Strongly agree

37. When I see someone injured or in need, I feel a strong urge to take care of them:

Strongly disagree 1 2 3 4 5 6 7 Strongly agree

38. Taking care of others warms my heart:

Strongly disagree 1 2 3 4 5 6 7 Strongly agree

39. I often notice people who need help:

Strongly disagree 1 2 3 4 5 6 7 Strongly agree

40. I am a very compassionate person:

Strongly disagree 1 2 3 4 5 6 7 Strongly agree

41. You are :

- A man
- A woman

42. How old are you ?

- <25 years old
- 25-40 years old
- 41-50 years old
- 51-60 years old
- >60 years old

43. What is your occupation? (single choice)

- Healthcare assistant
- Childcare assistant
- Maintenance worker
- Stretcher Bearer
- Nurse manager
- Students (nurses, healthcare assistant, medical student …)
- Nurse
- Resident
- Physiotherapist
- Physician
- Pharmacist
- Laboratory staff
- Pharmacy staff
- Clinical research staff
- Midwife
- Technical agent
- Other paramedic (social worker, psychologist, occupational therapist ...)
- Medical secretary
- Adminisatrative manager
- Administrative agent

- Other: ……………………

44. What is your education level?

- No degree
- College certificate
- BEP/CAP
- Baccalaureate
- BTS/DUT
- BAC + 3
- BAC + 4
- ≥ BAC+5
- Other: ……………………………………………………………

45. If you are a physician, midwife or pharmacist, will you recommend the vaccine to your patients, following the recommendations that will be made?

- Yes, of course
- Yes, maybe
- No
- I don't know

46. What is the status of your healthcare facility?

- Public
- Private

47. What is the type of your healthcare facility?

- Hospitall
- Clinic
- Nursing home
- Other

48. Is seasonal flu vaccination offered by your facility?

- Yes
- No
- I don’t know

49. How do you think access to Covid-19 vaccination could be made easier at your facility? (multiple responses possible):

- Same system as for the flu vaccination: provision of the vaccine in the department with a referent or at the occupational medicine
- Nominative provision of the vaccine by my institution to my primary care physician
- Provision of vaccine during an in-facility campaign with group vaccination sessions
- Do you have any other ideas? …………………………………………………………………

50. How do you think access to Covid-19 vaccination could be made easier in your city? (multiple responses possible):

- Same system as for the flu vaccination: provision of the vaccine in pharmacies by name
- Nominative provision of the vaccine in primary care physicians
- Provision of the vaccine through a nominative vaccination campaign: collective vaccination sessions
- Do you have any other ideas? …………………………………………………………………

51. Consider that this scale represents all employees in your facility. At the top of the scale are those who are most advantaged, at the bottom are those who are least advantaged

Select the number where you think you stand according to your income, your degree(s), your professional status:

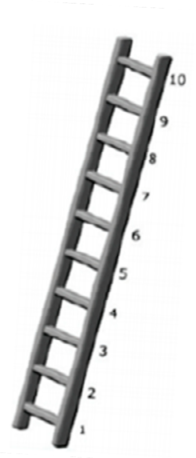
10
9
8
7
6
5
4
3
2
1
